# Biomolecule-assisted synthesis of carbon nitride and sulfur-doped carbon nitride heterojunction nanosheets: An efficient heterojunction photocatalyst for photoelectrochemical applications

**DOI:** 10.3762/bjnano.5.89

**Published:** 2014-06-03

**Authors:** Hua Bing Tao, Hong Bin Yang, Jiazang Chen, Jianwei Miao, Bin Liu

**Affiliations:** 1 School of Chemical and Biomedical Engineering, Nanyang Technological University, 62 Nanyang Drive, Singapore 637459, Singapore

**Keywords:** graphitic carbon nitride (g-C_3_N_4_), heterojunction, photoelectrochemical, photocatalysis, sulfur doping

## Abstract

A biomolecule-assisted pyrolysis method has been developed to synthesize sulfur-doped graphitic carbon nitride (CNS) nanosheets. During the synthesis, sulfur could be introduced as a dopant into the lattice of carbon nitride (CN). Sulfur doping changed the texture as well as relative band positions of CN. By growing CN on preformed sulfur-doped CN nanosheets, composite CN/CNS heterojunction nanosheets were constructed, which significantly enhanced the photoelectrochemical performance as compared with various control counterparts including CN, CNS and physically mixed CN and CNS (CN+CNS). The enhanced photoelectrochemical performance of CN/CNS heterojunction nanosheets could be ascribed to the efficient separation of photoexcited charge carriers across the heterojunction interface. The strategy of designing and preparing CN/CNS heterojunction photocatalysts in this work can open up new directions for the construction of all CN-based heterojunction photocatalysts.

## Introduction

Over the past few years, graphitic carbon nitride (CN) has attracted significant research attention in visible-light-driven photocatalysis because of its unique physical and chemical properties including chemical and thermal stability, physical abundance, as well as suitable bandgap energy and band position [1–4]. The polymeric nature of CN could facilitate the tuning of the physical and chemical properties by simply changing the CN precursors, by varying the pyrolysis conditions and by doping with foreign atoms [5–8]. However, the photocatalytic performance of CN is still limited because of the fast charge recombination [6,8–10]. How to efficiently separate photogenerated charge carriers in CN becomes a critical factor in further improving the photocatalytic performance. The construction of heterojunctions is a simple and effective way to enhance charge carrier separation, in which the build-in electric field across the junction could drive electrons and holes moving towards different parts of the photocatalyst, and thus improving the lifetime of charge carriers [11]. Numerous CN-based heterojunctions have been constructed by coupling CN with various types of photocatalysts, e.g., oxides and chalcogenides, which have shown improved photocatalytic performances [12–18]. However, the formation of interfacial defects at the CN/photocatalyst heterojunction arising from lattice mismatches would trap photogenerated electrons or holes, which reduces the benefits of build-in electric field created from the heterojunction. A smooth transition from one crystal phase to the other in a heterojunction could minimize the formation of interfacial defects, thus benefiting the interfacial charge transfer [19]. The formation of a smooth crystal transition would be expected at the interface of an all CN-based heterojunction. However, it is still challenging to synthesize a composite CN photocatalyst which is solely based on CN with different band structures [20].

Herein, we employ a biomolecule-assisted (L-cysteine) pyrolysis method to synthesize sulfur-doped carbon nitride (CNS) nanosheets, which can serve as the framework to grow CN to form an all CN-based heterojunction composite. The formation of CN/CNS heterojunctions significantly improves the photoelectrochemical performance, which is attributed to the efficient separation of photoexcited charge carriers across the heterojunction interface. The strategy of designing and preparing CN/CNS heterojunction photocatalysts in this work can open up new directions for the construction of all CN-based heterojunction photocatalysts.

## Results and Discussion

**Figure 1:**
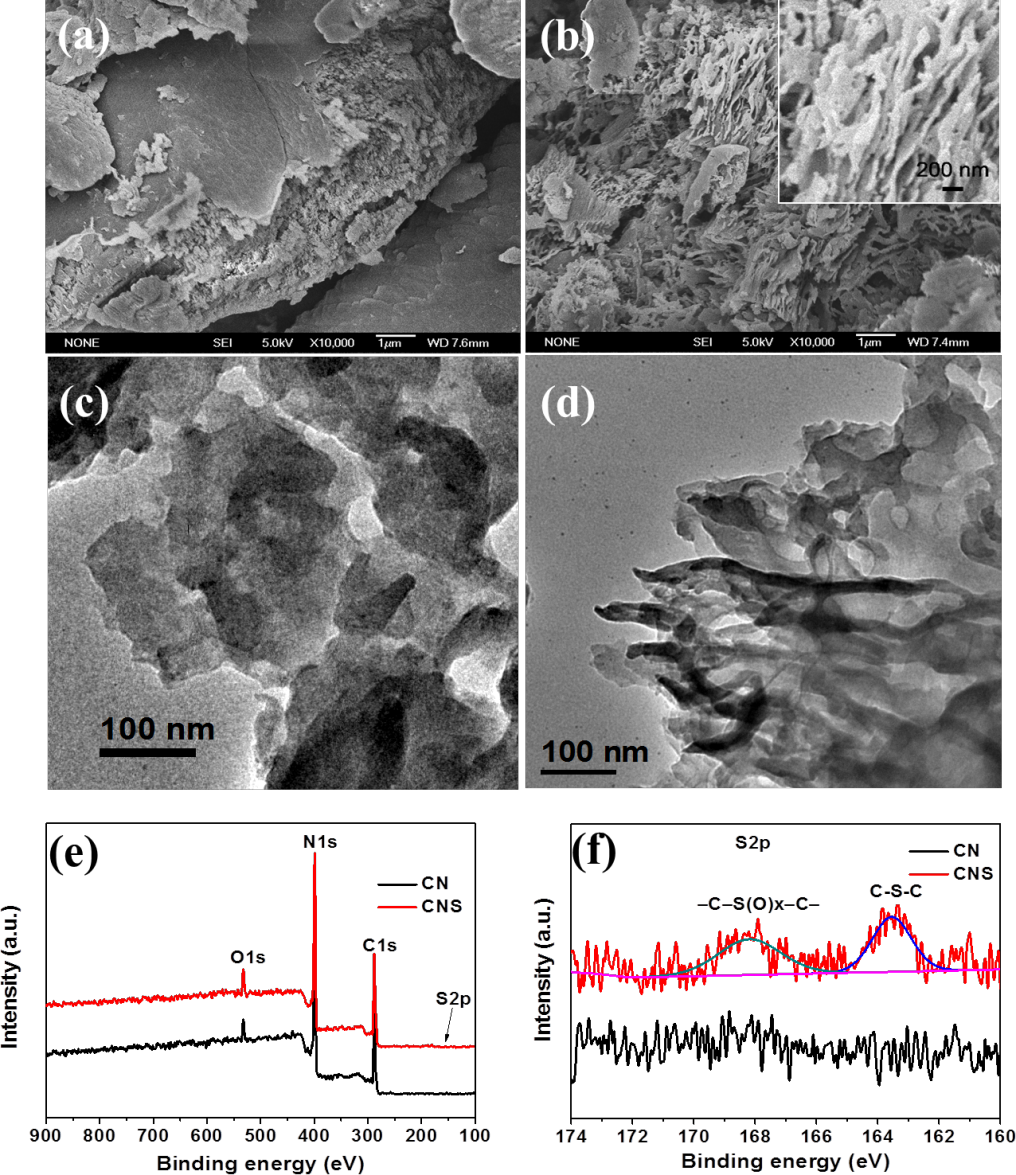
FESEM and TEM images of (a,c) CN, and (b,d) CNS samples. The inset of (b) is an enlarged FESEM image of the CNS sample. (e,f) Survey XPS spectra and high-resolution S2p XPS spectra of CN and CNS samples.

Figure 1a and Figure 1b show the FESEM images of as-prepared CN and CNS. CN is composed of large micrometer-sized particles, whereas CNS consists of small pieces of thin nanosheets, which are loosely connected with each other. Transmission electron microscopy gives a deeper insight into the crystal structure and morphology. As shown in Figure 1c and Figure 1d, in stark contrast with CN, CNS nanosheets are much smaller and thinner with worm-like nanopores dispersed on the surface of the nanosheets, owing predominantly to the drastic decomposition/delamination of CNS at high temperature. X-ray photoelectron spectroscopy (XPS) measurements were performed to study the chemical states of CN and CNS. As shown in the survey spectra in Figure 1e, CN has the typical C1s and N1s signals, which evidence the successful formation of CN through thermal decomposition and polymerization of the amine precursor. Besides C1s and N1s signals, an additional S2p signal is observed in CNS. The atomic concentration of sulfur in CNS is determined to be about 0.36 atom %. The high-resolution S2p spectrum of CNS deconvolutes into two peaks centered at 163.5 and 168.2 eV, which could be ascribed to S–(C)_3_ (tertiary sulfur) and C–S(O)–C (secondary sulfur), respectively, providing a compelling evidence of sulfur doping (sulfur is introduced into CN through substituting lattice nitrogen with sulfur on both tertiary and secondary nitrogen sites) [21]. The higher intensity for the peak centered at 163.5 eV than for that at 168.2 eV suggests that the replacement of the secondary nitrogen with sulfur in CN is more favorable.

**Figure 2:**
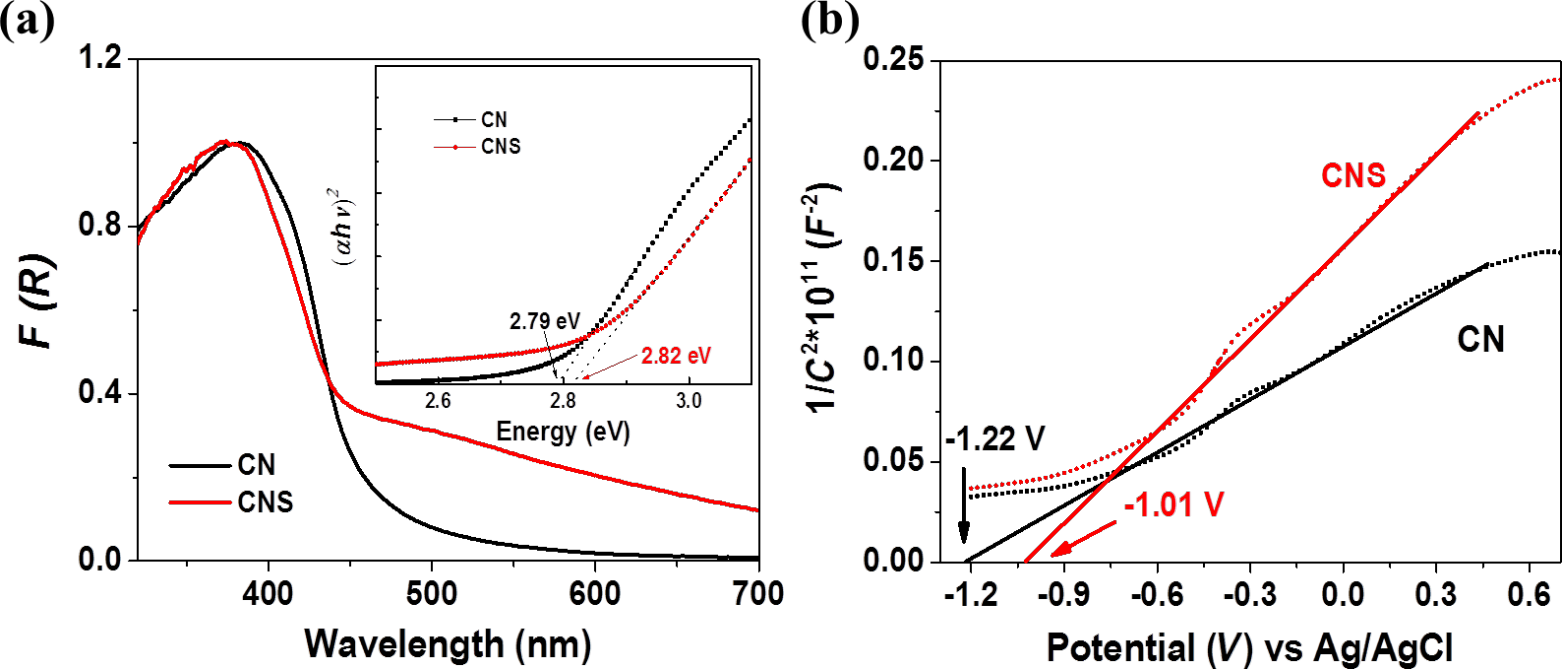
(a) Optical absorption spectra of CN and CNS. Inset shows the Tauc plots for bandgap determination, (b) Mott–Schottky plots for CN and CNS.

The effects of sulfur doping on the optical and energy band structure were investigated by UV–vis absorption and Mott–Schottky measurements. Figure 2a shows the UV–vis diffuse reflectance spectra of CN and CNS. Both CN and CNS feature a semiconductor-like absorption. The abrupt absorption onset for CN and CNS at ca. 450 nm is due to the photoexcitation of electrons from the valence band to the conduction band. The tail absorption in the long wavelength region for CNS could be attributed to the interband transition induced by defects through sulfur doping. The bandgap energy (*E*_g_) estimated from the (α*hν*)^2^ versus *hν* plots are 2.79 and 2.82 eV for CN and CNS, respectively. Mott–Schottky measurements were conducted to estimate the relative conduction band position. From the intersects of the Mott–Schottky plots, the flatband potential and thus the conduction band edge of CN and CNS are estimated to be about −1.22 and −1.01 eV vs Ag/AgCl, respectively. Together with the bandgap energy obtained from optical absorption measurements, the valence band position for CN and CNS are estimated to be about 1.57 and 1.81 eV vs Ag/AgCl.

**Figure 3:**
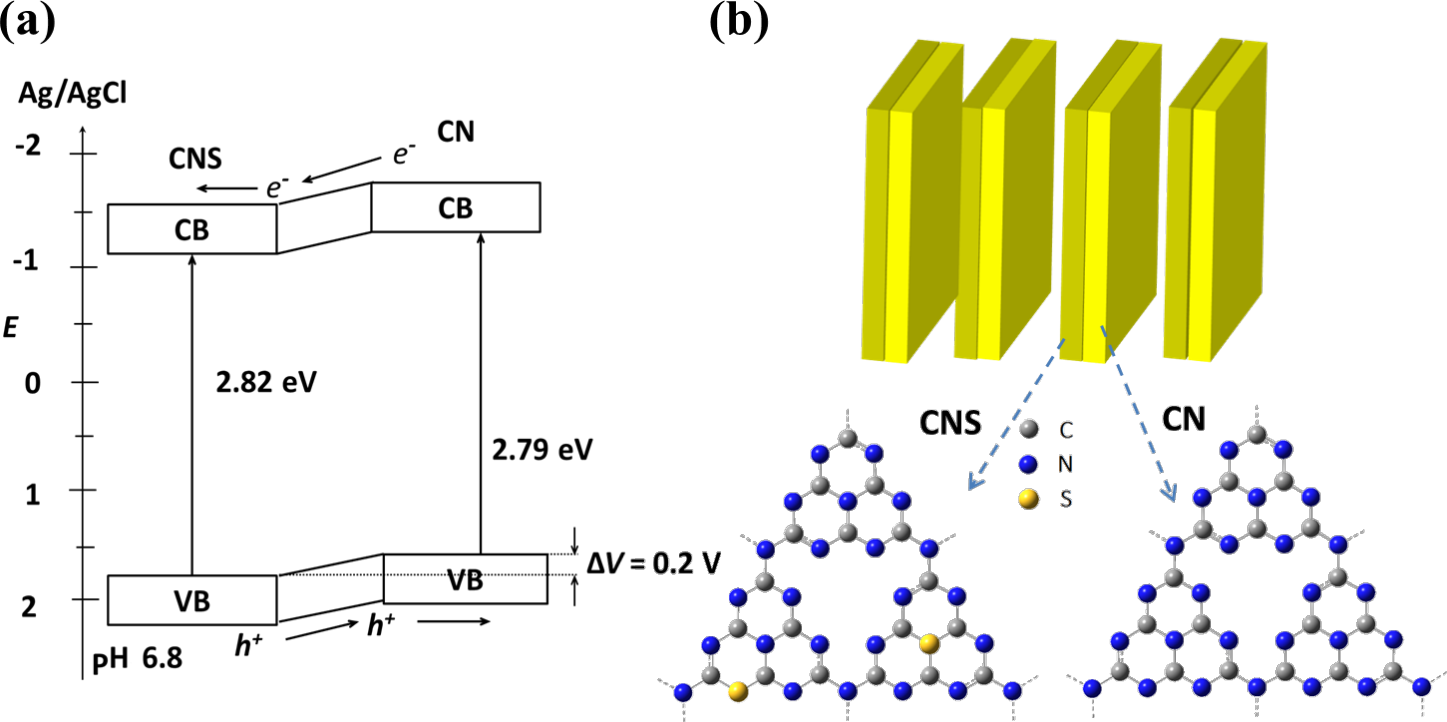
Schematic illustration of (a) electron–hole separation at the CN/CNS heterojunction interface, (b) the structure of CN/CNS heterojunction.

Based on the above analysis, it is clear that CN can form a type-II heterojunction upon CNS with band offsets of 0.24 eV (1.57 eV/1.81 eV vs Ag/AgCl) and 0.21 eV (−1.22 eV/−1.01 eV vs Ag/AgCl) at the valence and conduction band, respectively (Figure 3a). This type-II band alignment means that once CN and CNS are electronically coupled, a well-matched band structure for charge separation will be formed. In this case, the photogenerated electrons are transferred from CN to CNS, while the photogenerated holes are transferred from CNS to CN, leading to an improved charge separation. To test our hypothesis, we designed a strategy to construct CN/CNS heterostructures. In our method, we firstly grow CNS nanosheets by using a biomolecule-assisted pyrolysis method, followed by growing CN on preformed CNS nanosheets to form a well-mixed CN/CNS heterostructure.

**Figure 4:**
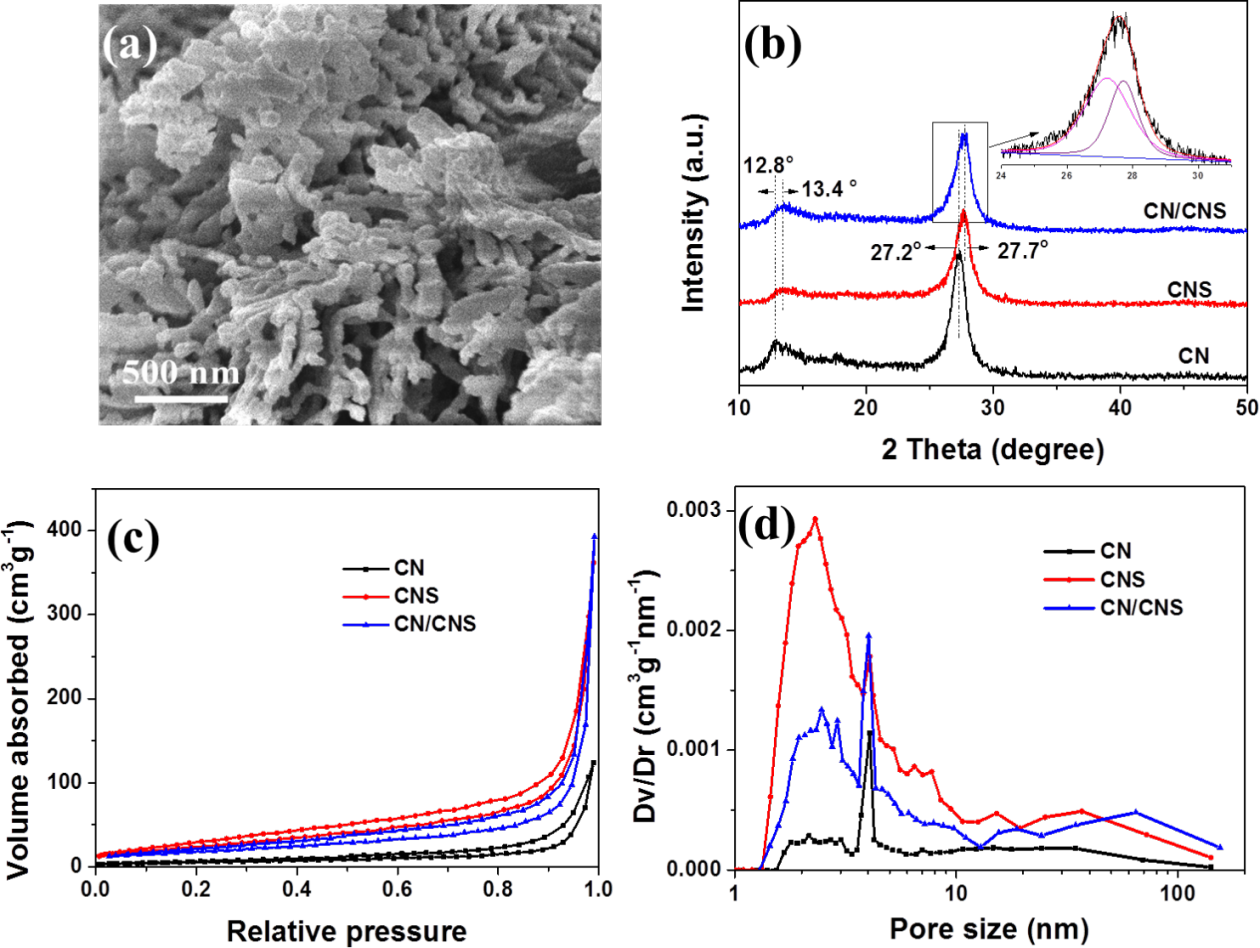
(a) FESEM image of CN/CNS heterostructure, (b) XRD and (c) nitrogen adsorption–desorption isotherms and (d) pore size distribution (insert) of CN, CNS and CN/CNS heterostructure.

Figure 4a shows the FESEM image of a CN/CNS composite heterostructure. In comparison with CNS (Figure 1b), the framework of CNS was well preserved after CN growth, while the thickness of nanosheets increased from ca. 30 nm for CNS to ca. 50 nm for CN/CNS composite. Figure 4b shows the XRD patterns of CN, CNS and the CN/CNS heterojunction composite. Two diffraction peaks are clearly visible in all XRD patterns. The peak centered at 13° could be indexed to the (100) plane of CN, corresponding to the in-plane structural packing motif of tristriazine unit. The peak was slightly shifted towards a higher diffraction angle when sulfur was introduced into the CN lattice. The calculated hole-to-hole distances in the nitride pores of CN and CNS are 0.691 and 0.660 nm, respectively. The other peak at 27.5° could be assigned to the interlayer stacking of aromatic units, which is also noted as (002) plane. Analogous to the (100) peak, the (002) peak shifted from 27.2° in CN to 27.7° in CNS, indicating a reduction of the inter-plane distance after sulfur doping. The calculated interlayer distances of CN and CNS are 0.327 and 0.322 nm, respectively. The (002) peak of CN/CNS becomes wider than that of CN and CNS. A further analysis shows that the peak of CN/CNS can be split into two peaks at 27.2° and 27.7°, corresponding to the (002) peaks of CN and CNS, respectively. The composite peak of CN/CNS indicates that CN/CNS is a hybrid of CN and CNS, confirming the formation of a CN/CNS heterostructure. Nitrogen adsorption–desorption analysis in Figure 4c confirmed the significant change of the CN texture after sulfur doping. The specific surface area for CN and CNS are 21 and 118 cm^2^·mg^−1^, respectively. The surface area of the CN/CNS heterostructure (about 56 cm^2^·mg^−1^) is reduced to about half of that of CNS due to increased thickness of nanosheets. Figure 4d presents the pore size distribution of CN, CNS and the CN/CNS heterostructure. It is clear that sulfur doping significantly increases the pore volume of micro- (smaller than 10 nm) and meso- (larger than 100 nm) pores, which could favor the photocatalytic performance [22].

**Figure 5:**
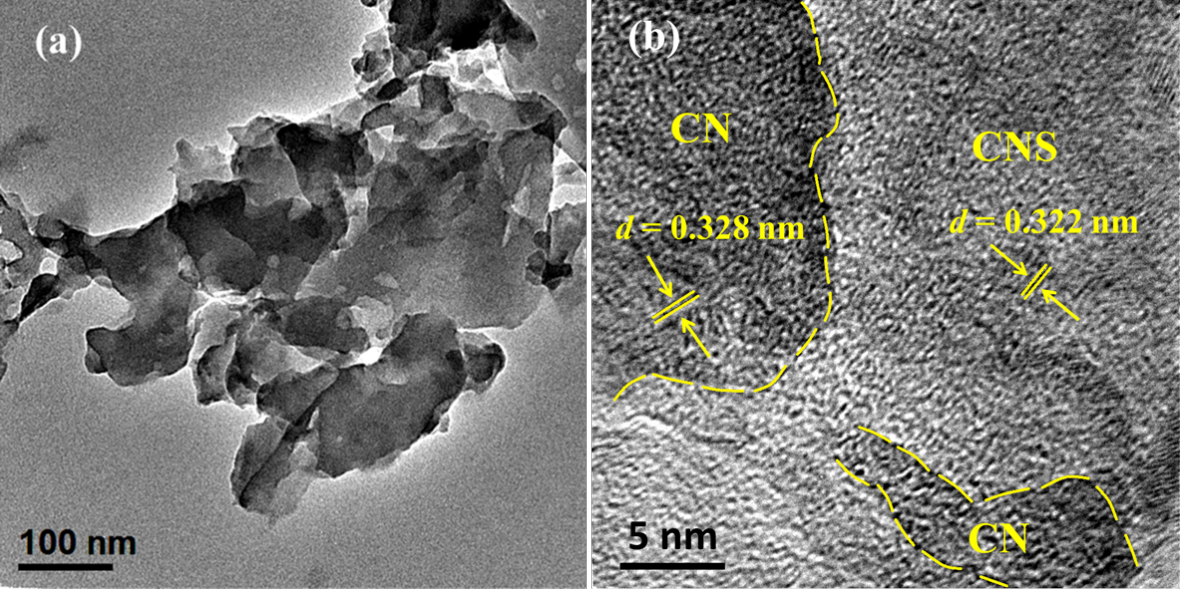
TEM (a) and HRTEM (b) images of a CN/CNS heterostructure.

Figure 5 shows the TEM image of CN/CNS heterostructure. As shown in Figure 5a, a layered structure made up of a dark region and a dim region can be clearly distinguished. The dark and dim region can be ascribed to dense CN and CNS nanosheets, respectively, based on the high-resolution TEM (HRTEM) analysis as shown in Figure 5b. Furthermore, it can be observed that the dense CN layer intimately connects with the CNS nanosheet to form a heterostructure. The HRTEM image of CN/CNS as shown in Figure 5b clearly distinguishes the phases of CN (the dark region) and CNS (the dim region). The lattice spacing for the dark region and the dim region are 0.328 nm and 0.322 nm respectively, which are consistent with the XRD results. The HRTEM image gives solid evidence towards the formation of heterojunction in CN/CNS.

**Figure 6:**
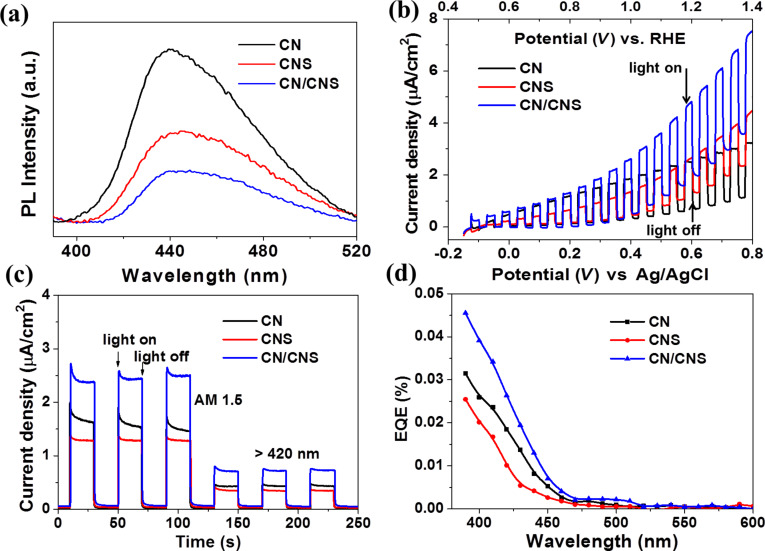
(a) Photoluminescence of CN, CNS and CN/CNS in aqueous solution. (b) Current density–voltage (*J*–*V*) curves for CN, CNS and CN/CNS electrodes under simulated sunlight (AM 1.5, 100 mW/cm^2^). All experiments were performed in 0.2 M Na_2_SO_4_ aqueous solution at a scan rate of 10 mV/s. (c) Current density–time (*J*–*t*) curves for CN, CNS and CN/CNS electrodes with 0.4 V bias vs Ag/AgCl under simulated sunlight (AM 1.5, 100 mW/cm^2^) and visible light (λ > 420 nm). (d) External quantum efficiency (EQE) of CN, CNS and CN/CNS photoelectrodes.

Figure 6a shows the photoluminescence (PL) spectra of CN, CNS and a CN/CNS heterostructure. In comparison with CN and CNS, a substantial reduction in PL intensity was observed for the hybrid CN/CNS heterostructure, suggesting an efficient charge carrier separation at the CN/CNS interface. Linear sweep photovoltammetry measurements were performed to study the photoelectrochemical performances of CN, CNS and the CN/CNS heterostructure. As shown in Figure 6b, photocurrent densities of the samples increase with forward bias voltage, indicating a typical n-type semiconductor behavior. Among all samples, the CN/CNS heterostructure demonstrates the highest photocurrent as compared with the other counterparts including CN, CNS and physically mixed CN and CNS over the entire potential profile. It is worth noting that the dark current densities of the photoelectrodes follow the order of CN/CNS heterostructure > CNS > CN, indicating the best charge transport properties of CN/CNS photoelectrode, which can be attributed to the large contact area between the CN/CNS photoelectrode and the electrolyte as well as an appropriate band alignment of the CN/CNS interface. It has been well-established that photocurrent is generated because of the diffusion of photogenerated electrons to the back contact and the simultaneous consumption of photogenerated holes by the hole acceptor in the electrolyte. As such, the superior photocurrent of CN/CNS heterostructure indicates the more efficient charge carrier separation and longer lifetime of the free charge carriers. A control experiment using physically mixed CN and CNS (1:1 in mass, named as CN+CNS) as photoelectrode was preformed (UV–vis and *J*–*V* curves are shown in Figure S1a and Figure S1b, respectively). Although the absorption of physically mixed CN and CNS is nearly the same as that of the CN/CNS heterojunction sample, the photocurrent of CN+CNS is much lower, implying that the enhanced photoresponse of heterojunction sample comes from better separation of photogenerated electrons and holes instead of improved photon absorption. The mechanism for photocurrent enhancement was further studied by measuring the photoresponse under different light source and the EQE spectra. Figure 6c exhibits the photoresponse of CN, CNS and CN/CNS under different light sources. It can be observed that the photocurrent can be reproducibly produced under AM 1.5G simulated sunlight or visible light (λ > 420 nm) with the same trend following the order of CN/CNS heterostructure > CN > CNS. The low photocurrent density might be due to the poor contact among CN particles and FTO substrate. Figure 6d shows the external quantum efficiency (EQE) of CN, CNS and the CN/CNS heterostructure, which matches well with the corresponding photocurrent density. It is worth mentioning that the shape of the three EQE curves are similar with the same cut off at nearly 470 nm, indicating that the enhanced photocurrent of CN/CNS heterostructure mainly comes from improved charge separation at the CN/CNS heterojunction interface.

## Conclusion

In conclusion, we have developed a biomolecule-assisted pyrolysis method to synthesize sulfur doped carbon nitride nanosheets (CNS), which offers an effective way to modify the texture and energy band positions of carbon nitride (CN). By growing CN on preformed sulfur-doped CN nanosheets, composite CN/CNS heterojunction nanosheets were constructed, which exhibited a significantly enhanced photoelectrochemical performance as compared with various control counterparts including CN, CNS and physically mixed CN and CNS (CN+CNS). The enhanced photoelectrochemical performance of CN/CNS heterojunction nanosheets could be ascribed to the efficient separation of photoexcited charge carriers across the heterojunction interface. Our approach offers a facile way to construct an all carbon nitride based heterojunction photocatalyst.

## Experimental

### Materials preparation

Graphitic carbon nitride (CN) was prepared according to a reported pyrolysis method [23]. Typically, 2 g of melamine powder was put into an alumina crucible covered with a piece of titanium sheet, then heated at a heating rate of 2.3 °C/min to 550 °C in a tube furnace and maintained at this temperature for 4 h under flowing argon. To synthesize sulfur-doped carbon nitride (CNS), 222 mg of L-cysteine was blended with 2 g of melamine in an agate mortar, wherein L-cysteine acts as the sulfur source for sulfur doping. During the pyrolysis process, the –SH functional group in L-cysteine reacts with the amine group in melamine to substitute the N atoms and to form the S–C bond. Following, this mixture was heated to 550 °C at a heating rate of 2.3 °C/min and maintained at this temperature for 2 h. The CN/CNS heterojunction was prepared through thermal condensation of melamine on preformed CNS nanosheets. Specifically, 1 g of melamine and 1 g of preformed CNS nanosheets were mixed together and underwent the same pyrolysis process as that for the preparation of CN. Physical mixtures of CN and CNS (1:1 mass ratio) were also prepared as reference.

### Characterization

The morphology of the samples was examined by using ﬁeld emission scanning electron microscopy (FESEM, JEOL, JSM6701F) and transmission electron microscopy (TEM, JEOL 3010). The chemical bonding information was studied with X-ray photoelectron spectroscopy (Kratos AXIS Ultra spectrometer) with a monochromatized Al Kα X-ray source (1486.71 eV). The Brunauer–Emmett–Teller (BET) surface area of sample was obtained on a nitrogen adsorption apparatus (Autosorb-6B, Quantachrome Instruments) with all samples degassed at 150 °C for 16 h prior to the measurement. The UV–vis diffuse reﬂectance spectra (DRS) were obtained on a UV–vis spectrometer (ShimadzuUV2450) using BaSO_4_ as reference. The powder X-ray diffraction (XRD) patterns were obtained on a Bruker D2 diffractometer (Bruker AXS, λ = 0.15418 nm). The chemical states and percentage of sulfur were measured by using X-ray photoelectron spectroscopy (XPS) on a VG Escalab 220i XL and the binding energies were calibrated by using the C 1s peak at 285.0 eV. The photoluminescence (PL) spectra were recorded using an LP920-KS instrument from Edinburgh Instruments, equipped with a photomultiplier tube.

### Photoelectrochemical measurements

The photoelectrochemical properties of as-prepared samples were measured by using an electrochemical workstation (CHI 760E, CH Instrument Inc., USA) in a standard three-electrode setup with a Pt plate as the counter electrode and an Ag/AgCl as the reference electrode. In all cases, 0.2 M Na_2_SO_4_ aqueous solution (pH 6.8) was used as the electrolyte. Prior to each measurement, the electrolyte was deaerated by continuously purging nitrogen for 30 min. The working electrode was prepared as the following: briefly, 10 mg of as-prepared sample was suspended in 1 mL of isopropyl alcohol (IPA). Then, 50 μL of the colloidal suspension (10 mg/mL) was dropcasted onto precleaned fluorine-doped tin oxide (FTO) substrate with a fixed area of 1 cm^2^. After coating, the film was dried at 70 °C in ambient atmosphere, followed by annealing at 350 °C for 1 h under argon to improve physical and electrical contact. The light source used for photoelectrochemical measurement was a 300 W xenon lamp (Newport, Oriel, 91160) equipped with an AM 1.5G filter (Newport, 81094) and a UV-filter (Newport, FSQ-GG420) (cut off: 420 nm). Prior to each measurement, the light intensity was determined by a calibrated silicon photodiode. The external quantum efficiency (EQE) was measured under +0.4 V external bias (three-electrode) condition. The monochromatic light was supplied by a xenon lamp (300 W, Oriel) illuminating through a monochromator (Newport) with a bandwidth of 5 nm.

## Supporting Information

File 1 Additional experimental data.
