# Supplementary material for: Biomolecule-assisted synthesis of carbon nitride and sulfur-doped carbon nitride heterojunction nanosheets: An efficient heterojunction photocatalyst for photoelectrochemical applications
Source: Beilstein J Nanotechnol. 2014 Jun 3;5:770–7. doi: 10.3762/bjnano.5.89 (PMC4077510; doi:10.3762/bjnano.5.89)
Supplement: File 1 — Additional experimental data. [file Beilstein_J_Nanotechnol-05-770-s001.pdf]

Supporting Information

for

Biomolecule-assisted synthesis of carbon nitride and sulfur-doped carbon nitride heterojunction nanosheets: An efficient heterojunction photocatalyst for photoelectrochemical applications

Hua Bing Tao, Hong Bin Yang*, Jiazang Chen, Jianwei Miao and Bin Liu*

Address: School of Chemical and Biomedical Engineering, Nanyang Technological University, 62 Nanyang Drive, Singapore 637459, Singapore

Emails: Hong Bin Yang* - hbyang@ntu.edu.sg; Bin Liu* - liubin@ntu.edu.sg

* Corresponding author

Additional experimental data

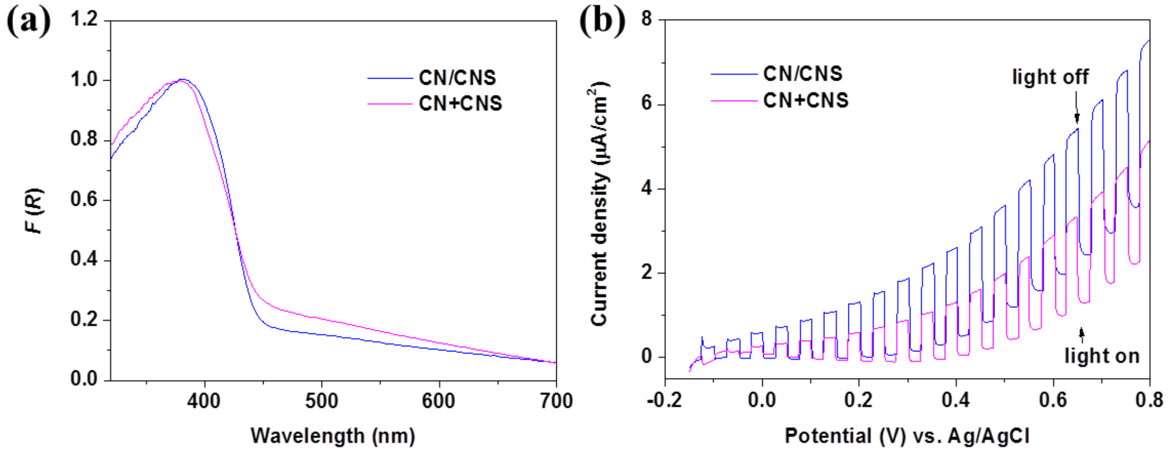

Figure S1: (a) Optical absorption spectra of CN/CNS and CN+CNS samples. (b) Current density–voltage (*J*–*V*) curves for CN/CNS and CN+CNS electrodes under simulated sunlight (AM 1.5, 100 mW/cm^2). All experiments were performed in 0.2 M Na_2SO_4 aqueous solution at a scan rate of 10 mV/s.

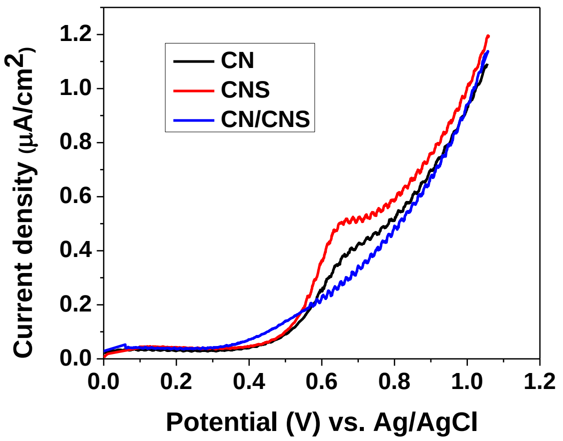

Figure S2: Linear Sweep Voltammetry of CN, CNS and CN/CNS electrodes under dark condition. All experiments were performed in 0.2 M Na_2SO_4 aqueous solution at a scan rate of 10 mV/s.
